# A copula method for modeling directional dependence of genes

**DOI:** 10.1186/1471-2105-9-225

**Published:** 2008-05-01

**Authors:** Jong-Min Kim, Yoon-Sung Jung, Engin A Sungur, Kap-Hoon Han, Changyi Park, Insuk Sohn

**Affiliations:** 1 Division of Science and Mathematics, University of Minnesota, Morris, MN, 56267, USA; 2 Department of Statistics, Kansas State University, Manhattan, Kansas, 66506, USA; 3 Department of Pharmaceutical Engineering, Woosuk University, Wanju, Jeonbuk, 565-701, Republic of Korea; 4 Department of Statistics, University of Seoul, Seoul 136-743, Republic of Korea; 5 Department of Statistics, Korea University, Seoul 136-701, Republic of Korea

## Abstract

**Background:**

Genes interact with each other as basic building blocks of life, forming a complicated network. The relationship between groups of genes with different functions can be represented as gene networks. With the deposition of huge microarray data sets in public domains, study on gene networking is now possible. In recent years, there has been an increasing interest in the reconstruction of gene networks from gene expression data. Recent work includes linear models, Boolean network models, and Bayesian networks. Among them, Bayesian networks seem to be the most effective in constructing gene networks. A major problem with the Bayesian network approach is the excessive computational time. This problem is due to the interactive feature of the method that requires large search space. Since fitting a model by using the copulas does not require iterations, elicitation of the priors, and complicated calculations of posterior distributions, the need for reference to extensive search spaces can be eliminated leading to manageable computational affords. Bayesian network approach produces a discretely expression of conditional probabilities. Discreteness of the characteristics is not required in the copula approach which involves use of uniform representation of the continuous random variables. Our method is able to overcome the limitation of Bayesian network method for gene-gene interaction, i.e. information loss due to binary transformation.

**Results:**

We analyzed the gene interactions for two gene data sets (one group is eight histone genes and the other group is 19 genes which include DNA polymerases, DNA helicase, type B cyclin genes, DNA primases, radiation sensitive genes, repaire related genes, replication protein A encoding gene, DNA replication initiation factor, securin gene, nucleosome assembly factor, and a subunit of the cohesin complex) by adopting a measure of directional dependence based on a copula function. We have compared our results with those from other methods in the literature. Although microarray results show a transcriptional co-regulation pattern and do not imply that the gene products are physically interactive, this tight genetic connection may suggest that each gene product has either direct or indirect connections between the other gene products. Indeed, recent comprehensive analysis of a protein interaction map revealed that those histone genes are physically connected with each other, supporting the results obtained by our method.

**Conclusion:**

The results illustrate that our method can be an alternative to Bayesian networks in modeling gene interactions. One advantage of our approach is that dependence between genes is not assumed to be linear. Another advantage is that our approach can detect directional dependence. We expect that our study may help to design artificial drug candidates, which can block or activate biologically meaningful pathways. Moreover, our copula approach can be extended to investigate the effects of local environments on protein-protein interactions. The copula mutual information approach will help to propose the new variant of ARACNE (Algorithm for the Reconstruction of Accurate Cellular Networks): an algorithm for the reconstruction of gene regulatory networks.

## Background

Genes interact with each other as basic building blocks of life, forming a complicated network. The relationship between groups of genes with different functions can be represented as gene networks. Recent developments in microarray technology revolutionized research in the life sciences, allowing researchers to measure tens of thousands of genes simultaneously [1,2]. With the deposition of huge microarray data sets in public domains, study on gene networking is now possible. Reconstructing gene networks from the microarray data will facilitate cellular function dissection at the molecular level. Hence the study will have a profound impact on biomedical research, ranging from cancer research to disease prevention [3].

There has been an increasing interest in the reconstruction of gene networks from gene expression data. Recent works include linear models [4,5], Boolean network models [6], and Bayesian networks [3,7-10]. Bayesian networks seem to be very effective in the construction of gene networks. They can incorporate prior knowledge from biology into their models and handle missing data effectively. In particular, dynamic Bayesian networks can learn a gene network from time-course gene expressions. As noted in [9], a major problem with Bayesian networks is the computation problem. Our motivation is to overcome this limitation of Bayesian networks in gene interactions. For this purpose, we introduce a simple method for constructing gene networks based on copulas. Note that copulas can model a variety of interactions.

In statistical literature, the general way to describe dependence between correlated random variables is to use copulas [11]. Copulas are multivariate distribution functions whose one-dimensional margins are uniform on the [0, 1] interval [12]. Copulas are useful for constructing joint distributions, especially with nonnormal random variables. The design, features, and some implementation details of the R package copula can be easily extended in multivariate modeling in many fields [13]. In finance, copula functions are adopted to handle the interaction between the markets and risk factors in a flexible way [14]. In biology, a gaussian copula has been applied in quantitative trait linkage. Copulas play an important role in developing a unified likelihood framework to analyze discrete, continuous, and censored traits [15]. In principle, copulas can be used to model the joint distributions of any discrete or continuous gene and even mixed continuous and discrete genes. In [16], several measures of directional dependence in regression based on copula functions were proposed. Recently, a sieve maximum likelihood estimation procedure for semiparametric multivariate copula models has been proposed in [17]. The proposed estimation achieved efficiency gains in finite samples, especially when prior information of the marginal distribution is incorporated. In this paper, we adopt a measure of directional dependence to investigate the gene interactions for yeast cell cycle data. One advantage of our approach is that dependence between genes is not assumed to be linear. Moreover, our approach can detect directional dependence. Hence our approach can provide valuable biological information on the presence of directional dependence between genes.

## Results and Discussion

**Figure 1:**
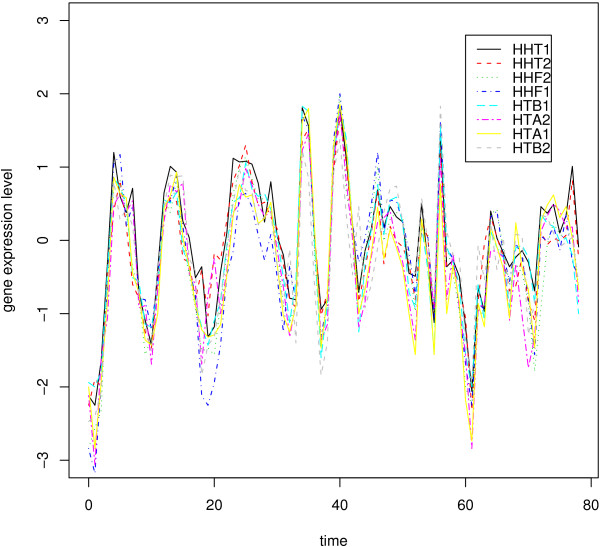
Time-series plot of gene expressions in histone group.

**Figure 2:**
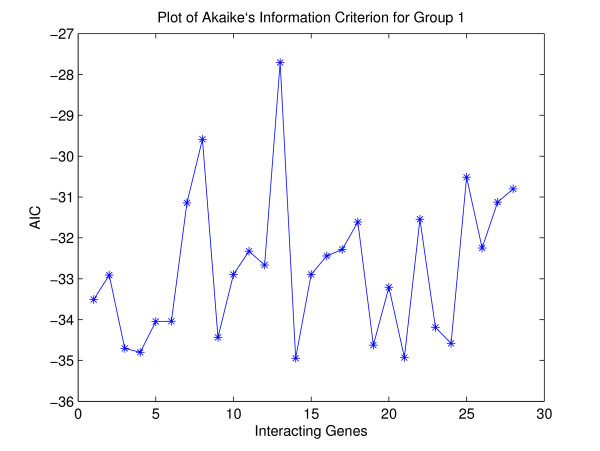
AIC plots for Table 1.

**Figure 3:**
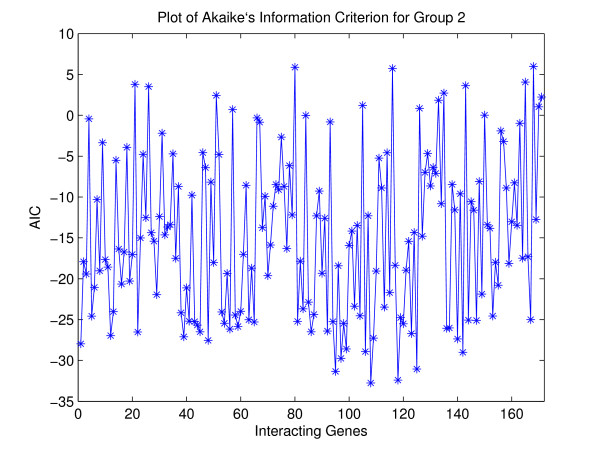
**AIC plots for **[see Additional file 1]**.**

In this section, we analyze yeast cell cycle regulation [18]. The data set is composed of measurements on 6221 genes observed at 80 time points. 800 genes regulated by cell cycle were identified. To compare our results with other results in the literature, we selected two groups of genes with known interaction patterns. Note that known interactions are still incomplete at present. The first group includes eight histone genes-HHT1, HHT2, HHF1, HHF2, HTA1, HTA2, HTB1 and HTB2. These eight genes encode for the four histones (H2A, H2B, H3 and H4). The histones are used to form the fundamental packaging unit of chromatin, called the code of nucleosome. Chromosomes, consisting of DNA and histones, need to be replicated before cell division. Expression of the histone genes should be regulated tightly for the proper functioning of the replication process. Figure 1 shows the time-series plot of genes in the histone group. It can be easily seen that the eight genes in the histone group are highly correlated with each other. Looking at Table 1 and Figure 2 for Group I dataset, we can find that those AIC values have pretty low values. It means that our copula method for group I dataset is appropriate. ¿From Figure 3 and [see Additional file 1] for Group II dataset, we also find that those AIC values have relatively inconsistent low values compared to Group I dataset. It still means that our copula method for group II dataset is also appropriate.

**Table 1:** Estimates of *α, β, θ *and proportions of variation for the directional dependence at Group I

		FGM type						Normal Type	
		
Interacting genes	AIC	$\hat{\alpha }$	$\hat{\beta }$	$\hat{\theta }$	$\rho_{C}^{2}$	$\rho_{U\to V}^{\left(2\right)}$	$\rho_{V\to U}^{\left(2\right)}$	$\bar{\theta_{*}}$	$\rho_{norm}^{2}$
HHT1 vs HHT2	-33.5084	1.0152	1.0199	1.0772	0.1048	0.1048	0.1048	0.832	0.67143
HHT1 vs HHF1	-32.9113	1.0152	1.0222	1.0772	0.1044	0.1044	0.1044	0.062	0.00350
HHT1 vs HHF2	-34.7051	1.0152	1.0229	1.0772	0.1042	0.1043	0.1043	0.163	0.02428
HHT1 vs HTA1	-34.7998	1.0152	1.0162	1.0772	0.1054	0.1054	0.1054	0.243	0.05411
HHT1 vs HTA2	-34.0483	1.0152	1.0218	1.0772	0.1044	0.1045	0.1045	0.135	0.01664
HHT1 vs HTB1	-34.0410	1.0152	1.0105	1.0567	0.1064	0.1064	0.1064	0.387	0.13831
HHT1 vs HTB2	-31.1447	1.0152	1.0234	1.0772	0.1042	0.1042	0.1042	0.217	0.04310

HHT2 vs HHF1	-29.5856	1.0199	1.0199	1.0964	0.1039	0.1040	0.1040	0.389	0.13976
HHT2 vs HHF2	-34.4365	1.0199	1.0229	1.0966	0.1034	0.1035	0.1035	0.752	0.54199
HHT2 vs HTA1	-32.8950	1.0199	1.0162	1.0814	0.1046	0.1046	0.1046	0.745	0.53143
HHT2 vs HTA2	-32.3277	1.0199	1.0218	1.0966	0.1036	0.1037	0.1037	0.968	0.93103
HHT2 vs HTB1	-32.6642	1.0199	1.0105	1.0567	0.1056	0.1056	0.1056	0.037	0.00124
HHT2 vs HTB2	-27.7089	1.0199	1.0234	1.0966	0.1033	0.1034	0.1034	0.935	0.86317

HHF1 vs HHF2	-34.9456	1.0222	1.0229	1.1055	0.1030	0.1031	0.1031	0.054	0.00265
HHF1 vs HTA1	-32.8945	1.0222	1.0162	1.0814	0.1042	0.1043	0.1042	0.721	0.49612
HHF1 vs HTA2	-32.4444	1.0222	1.0218	1.1041	0.1032	0.1033	0.1033	0.839	0.68354
HHF1 vs HTB1	-32.2815	1.0222	1.0105	1.0567	0.1052	0.1052	0.1052	0.178	0.02896
HHF1 vs HTB2	-31.6114	1.0222	1.0234	1.1055	0.1030	0.1030	0.1030	0.134	0.01639

HHF2 vs HTA1	-34.6262	1.0229	1.0162	1.0814	0.1041	0.1041	0.1041	0.508	0.24056
HHF2 vs HTA2	-33.2097	1.0229	1.0218	1.1041	0.1031	0.1032	0.1032	0.707	0.47617
HHF2 vs HTB1	-34.9285	1.0229	1.0105	1.0567	0.1051	0.1051	0.1051	0.203	0.03770
HHF2 vs HTB2	-31.5427	1.0229	1.0234	1.1083	0.1028	0.1029	0.1029	0.897	0.78898

HTA1 vs HTA2	-34.1910	1.0162	1.0218	1.0814	0.1042	0.1043	0.1043	0.847	0.69754
HTA1 vs HTB1	-34.5808	1.0162	1.0105	1.0567	0.1062	0.1063	0.1062	0.247	0.05591
HTA1 vs HTB2	-30.5148	1.0162	1.0234	1.0814	0.1040	0.1040	0.1041	0.389	0.13976

HTA2 vs HTB1	-32.2491	1.0218	1.0105	1.0567	0.1052	0.1053	0.1053	0.370	0.12628
HTA2 vs HTB2	-31.1265	1.0218	1.0234	1.1041	0.1030	0.1031	0.1031	0.256	0.06009

HTB1 vs HTB2	-30.8025	1.0218	1.0234	1.1004	0.1032	0.1032	0.1033	0.729	0.50774

**Figure 4:**
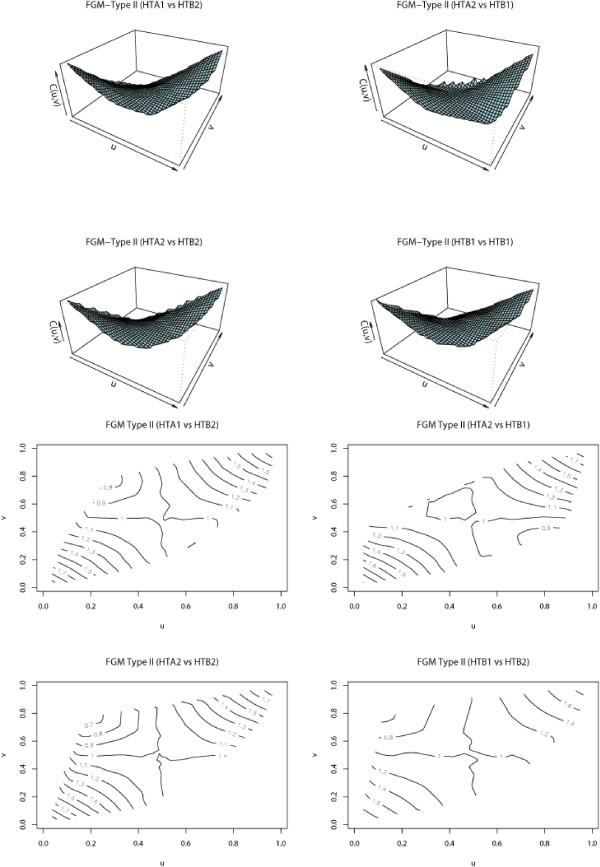
3D and contour plots for selected pairs of histone genes.

Because of the small number of gene data sets, the estimates of FGM parameters and proportions for directional dependence in Table 1 do not strongly support our claim that each pair of these 8 histone genes are dependent on each other in both directions. Figure 4 shows 3-dimensional and contour plots for HTA1 vs HTB2, HTA2 vs HTB1, HTA2 vs HTB2, and HTB1 vs HTB2. Irregularly shaped contours indicate the existence of directional dependence, i.e., the asymmetry of dependence. From the plots, we see that the asymmetry of dependence is not clear for each pair of genes. Contour plots for other pairs of histone genes show similar patterns. Figure 4 together with Table 1 tells us that the 3D and contour plots are relatively symmetric which means a weak directional dependence in this gene data set.

**Figure 5:**
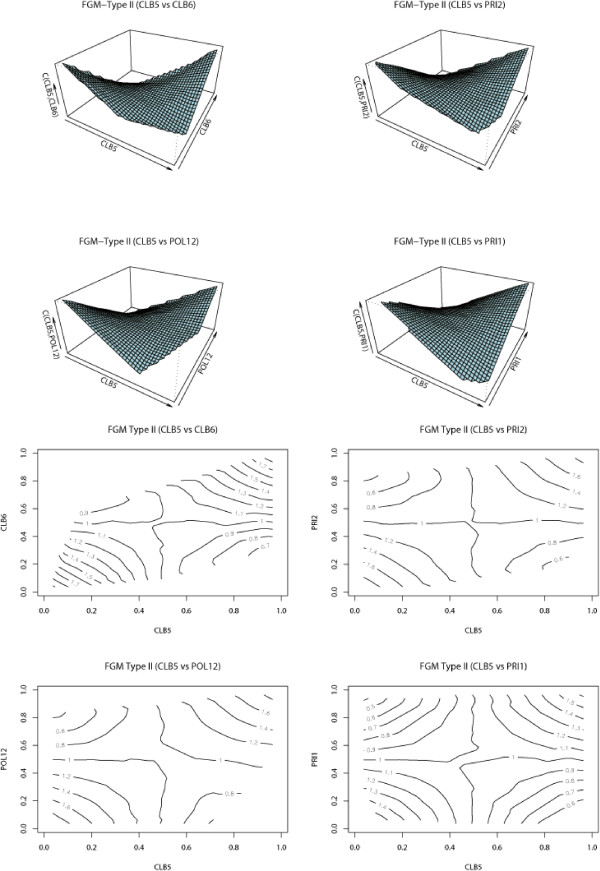
**3D and contour plots for selected pairs of histone genes **[see Additional file 1]**.**

**Figure 6:**
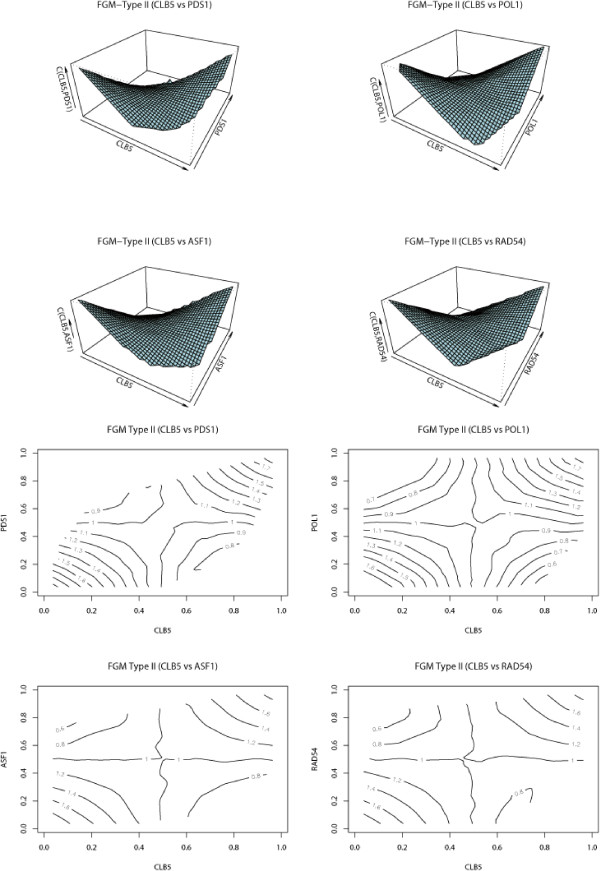
**3D and contour plots for selected pairs of histone genes** [see Additional file 1]**.**

To further evaluate the performance of the FGM copula model, we selected another group (Group II) which is comparatively larger than the first group. This group consisted of 19 genes which include DNA polymerases (POL1, POL2, POL12, and POL30), DNA helicase (HPR5), type B cyclin genes (CLB5 and CLB6), DNA primases (PRI1 and PRI2), radiation sensitive genes (RAD53 and RAD54), repaire related genes (MSH2, MSH6, and PMS1), replication protein A encoding gene (RFA3), DNA replication initiation factor (CDC45), securin gene (PDS1), nucleosome assembly factor (ASF1), and a subunit of the cohesin complex (MCD1). These genes play important role in the process of cell cycle which conducts DNA replication initiation, DNA damage-induced checkpoint arrest, DNA damage repair, formation of mitotic spindle, and so on. However, similar to the histone genes, their expression is also strictly regulated for the normal cellular process [19]. The estimates of FGM parameters and proportions for directional dependence [see Additional file 1] clearly support our claim that each pair of 19 genes are dependent on each other in both directions, which is consistent with the observation from Figure 5 and Figure 6.

Note that the measures of dependence $\rho_{C}^{2}$, $\rho_{U\to V}^{\left(2\right)}$, and $\rho_{V\to U}^{\left(2\right)}$ have different scales from usual correlation coefficient. Since Pearson's correlation coefficient is based on the assumption of normality and linearity of random variables *X *and *Y*, the range of Pearson's correlation is usually wider than that of our measures of directional dependence. Furthermore, Pearson's correlation coefficient depends on random variables *X *and *Y*, while the measures of directional dependence depend on the joint function of their cumulative distribution functions. Therefore, depending on the copula function adopted, the scales of the measures can be different. Also, when we use the uniform distribution or exponential distribution for the transformation of the marginal cumulative distribution functions of *X *and *Y*, the measure of dependence can be smaller than Pearson's Correlation coefficient. For a comparison of the measure of dependence of our FGM copula model, we used the normal copula model which is one of the representative copula models. If we look at the FGM type and Normal type in Table 1 and [see Additional file 1], we find that depending on the gene data pair, the measures of dependence using the normal copula has more variation then the measures of dependence using our proposed FGM copula. In light of these facts, our results are valid and consistent. To support our results, we also provided the matematical derivations of our proposed FGM copula model in the method section.

The results from our method have been compared with those from other methods such as PathwayAssist and Chen's method [3]. PathwayAssist (version 3.0) is based on a comprehensive gene (or protein) interaction database compiled by a text mining tool from the entire PubMed [20]. Our method found 28 edges among these 8 genes. From Table 2, we find that a PathwayAssist search identified 13 edges and Chen's method identified 12 edges. However, because two copies of each core histone i.e., H2A, H2B, H3 and H4, are assembled into an octamer, all 8 core histones can interact with each other. The 28 edges we found indicate that each histone gene is connected with the remaining 7 histone genes. All possible pairs of interaction genes from the group II [see Additional file 2]. The reason is that by using the FGM copula model, we are better able to investigate the better directional interaction dependence compared to PathwayAssist and Chen's method [3].

**Table 2:** Direct experimental support for the interactions uncovered

Interacting genes (locus name)	Our Method	PathwayAssist	Chen's method	Ref.
HTA2(YBL003C) – HTA1(YDR225W)	O	O	O	1
HTA2(YBL003C) – HHT2(YNL031C)	O	O	O	1
HTA2(YBL003C) – HTB2(YBL002W)	O	O	O	1
HTA2(YBL003C) – HHT1(YBR010W)	O	O	O	1
HTA2(YBL003C) – HHF1(YBR009C)	O	×	×	1
HTA2(YBL003C) – HHF2(YNL030W)	O	×	×	1
HTA2(YBL003C) – HTB1(YDR224C)	O	×	×	1

HTB2(YBL002W) – HTA1(YDR225W)	O	×	×	1
HTB2(YBL002W) – HHT2(YNL031C)	O	×	×	1
HTB2(YBL002W) – HHT1(YBR010W)	O	×	×	1
HTB2(YBL002W) – HHF1(YBR009C)	O	O	O	1
HTB2(YBL002W) – HHF2(YNL030W)	O	×	×	1
HTB2(YBL002W) – HTB1(YDR224C)	O	O	O	N.A.

HHT2(YNL031C) – HTA1(YDR225W)	O	×	O	N.A.
HHT2(YNL031C) – HHT1(YBR010W)	O	×	×	1
HHT2(YNL031C) – HHF1(YBR009C)	O	×	×	2
HHT2(YNL031C) – HHF2(YNL030W)	O	O	O	1
HHT2(YNL031C) – HTB1(YDR224C)	O	×	×	1

HHF1(YBR009C) – HTA1(YDR225W)	O	×	×	1
HHF1(YBR009C) – HHT1(YBR010W)	O	O	O	1
HHF1(YBR009C) – HHF2(YNL030W)	O	O	O	4
HHF1(YBR009C) – HTB1(YDR224C)	O	×	×	1

HHF2(YNL030W) – HTA1(YDR225W)	O	O	O	1
HHF2(YNL030W) – HHT1(YBR010W)	O	O	O	1
HHF2(YNL030W) – HTB1(YDR224C)	O	×	O	N.A.

HTA1(YDR225W) – HHT1(YBR010W)	O	×	×	1
HTA1(YDR225W) – HTB1(YDR224C)	O	O	O	2,3

HHT1(YBR010W) – HTB1(YDR224C)	O	×	×	1

Although microarray results show a transcriptional co-regulation pattern and do not imply that the gene products are physically interactive, this tight genetic connection may suggest that each gene product has either direct or indirect connections between the other gene products. Indeed, recent comprehensive analysis of a protein interaction map revealed that those histone genes are physically connected with each other [19], supporting the results obtained by our method. The findings of this study may help to design artificial drug candidates, which can block or activate biologically meaningful pathways. Furthermore, our copula approach can be extended to investigate the effects of local environments on protein-protein interactions. The copula mutual information approach will help to propose a new variant of ARACNE: an algorithm for the reconstruction of gene regulatory networks.

## Conclusion

In this paper, we presented a new methodology for analyzing gene interactions based on copula functions. Our method is shown to be useful in the construction of gene networks through the analysis of yeast cell cycle data. Our method may be able to overcome the limitation of Bayesian network method for gene-gene interaction, i.e. information loss due to binary transformation. Since a copula represents a way of extracting the dependence structure of the random variables from the joint distribution function, it is a useful approach to understanding and modeling dependent structure for random variables. In our future works on gene directional dependence, we will develop hypothesis testing for directional dependence and formulate a network construction process using false discovery rate.

## Methods

For presentation, let us consider a bivariate case. All the results in this section can be generalized to a multivariate case. Consider a bivariate copula *C *: [0, 1]^2^→ [0, 1] defined as

*C*(*u, v*) = *Pr*(*U *≤ *u, V *≤ *v*)

for 0 ≤ *u, v *≤ 1 where *U *and *V *are uniform random variables. Let *X *and *Y *be random variables with marginal distribution functions *F*_*X *_and *F*_*Y *_. Then *F*_*X *_(*X*) and *F*_*Y *_(*Y*) have uniform distributions. By Sklar's Theorem, due to [21], there exits a copula *C *such that *F *(*x, y*) = *C*(*F*_*X *_(*x*), *F*_*Y *_(*y*)) for all *x *and *y *in the domain of *F*_*X *_and *F*_*Y*_, i.e. a bivariate distribution function can be represented as a function of its marginals joined by a bivariate copula. Hence different families of copula correspond to different types of dependence structure. An example is the Farlie – Gumbel – Morgenstern class defined as *uv *[1 + *θ *(1 - *u*)(1 - *v*)] with *θ *≥ 0. See [12] for a general introduction to copulas.

Now we discuss the concept and measures of directional dependence briefly. One may consider two types of directional dependence between two random variables *U *and *V *in regression: *r*_*V*|*U *_(*u*) = *E*[*V*|*U *= *u*] and *r*_*U*|*V *_(*v*) = *E*[*U*|*V *= *v*] for the Rodrìguez-Lallena and Úbeda-Flores family of copula in the form of

(1) *C*(*u, v*) = *uv *+ *f*(*u*)*g*(*v*),

where *E*[*V*|*U *= *u*] is the conditional expectation of *V *given that *U *= *u *[22]. Note that a specific functional form of *f *and *g *determines the corresponding family of bivariate distributions of (*U, V*). If *f *and *g *are different, then the copula is not symmetric, in which case the form of the regression functions for *V *and *U *will be different. Hence one might consider two types of directional dependence, i.e. one in the direction from *U *to *V *and the other in the direction from *V *to *U*. Since directional dependence can arise from marginal or joint behavior or both, one may consider the following general measure of directional dependence defined as

(2) $$\begin{array}{lll} \rho_{X\to Y}^{\left(k\right)} & = & \frac{E{\left[r_{Y|X}(X)-E[Y]\right]}^{k}}{\mu_{k}(Y)}\text{ if }\mu_{k}(Y)=E{\left[Y-E[Y]\right]}^{k}\neq 0;\\ \rho_{Y\to X}^{\left(k\right)} & = & \frac{E{\left[r_{X|Y}(Y)-E[X]\right]}^{k}}{\mu_{k}(X)}\text{ if }\mu_{k}(X)\neq 0, \end{array}$$

where $\rho_{X\to Y}^{\left(k\right)}$ is the proportion of the *k*-th central moment of *Y *explained by the regression of *Y *on *X*. For example, $\rho_{X\to Y}^{\left(2\right)}$ can be interpreted as the proportion of variation explained by the regression of *Y *on *X *with respect to total variation of *Y*. For more details, see [16].

Finally, let us introduce the FGM distributions and measures of directional dependence for our data analysis. We consider the following type of FGM distributions in the form of the Rodrìguez-Lallena and Úbeda-Flores copula family in (1):

(3) *C*(*u, v*) = *uv *+ *θ uv*(1 - *u*)^*α *^(1 - *v*)^*β *^for 0 ≤ *u, v *= 1 and *α, β *≥ 1,

where *θ, α *and *β *are parameters. *C*(*u, v*) defined in (3) is a copula function for *θ *satisfying

(4) $$0\leq \theta \leq \min\left\{{\left(\frac{\alpha +1}{\alpha -1}\right)}^{\alpha -1},{\left(\frac{\beta +1}{\beta -1}\right)}^{\beta -1}\right\};$$

see [23].

Let *X*_*i *_and *Y*_*i *_be i.i.d. copies of *X *and *Y *for *i *= 1, ..., *n*. Then *U*_*i *_= *F*_*X *_(*X*_*i*_) and *V*_*i *_= *F*_*Y *_(*Y*_*i*_) are the empirical marginal distribution functions of *F*_*X *_and *F*_*Y*_. Note that *U*_*i *_and *V*_*i *_have uniform distributions on (0, 1). The empirical likelihood is

(5) $$L(\theta ;U,V)\propto \prod_{i=1}^{n}c(U_{i},V_{i}),$$

where **U **= (*U*_1_, ..., *U*_*n*_)*' *and **V **= (*V*_1_, ..., *V*_*n*_)*'*. From (3), the empirical likelihood function is

$$L(\theta ;u,v)\propto \prod_{i=1}^{n}\{1+\theta [1-u_{i}(1+\alpha )]{\left(1-u_{i}\right)}^{\alpha -1}[1-v_{i}(1+\beta )]{\left(1-v_{i}\right)}^{\beta -1}\}.$$

Solving

$$\frac{\partial \log L(\theta ;u,v)}{\partial \alpha }=0\text{ and }\frac{\partial \log L(\theta ;u,v)}{\partial \beta }=0$$

subject to *α, β *≥ 1, one obtains the estimates of *α *and *β *denoted by $\hat{\alpha }$ and $\hat{\beta }$. Since log *L*(*θ*; **u**, **v**) is a linear function of *θ *with known $\hat{\alpha }$ and $\hat{\beta }$, there is no closed form solution for MLE from the partial derivative function with respect to *θ*. As an alternative, we used a grid search over the range of *θ *with *α *= $\hat{\alpha }$ and *β *= $\hat{\beta }$.

For (3), we have $f(u)=\sqrt{\theta }u{\left(1-u\right)}^{\alpha }$ and $g(v)=\sqrt{\theta }v{\left(1-v\right)}^{\beta }$. The directional dependence from *U *to *V *and from *V *to *U *are given as

$$r_{U|V}(v)=\frac{1}{2}-\theta Beta(2,\alpha +1){\left(1-v\right)}^{\beta -1}[1-(1+\beta )v]$$

and

$$r_{V|U}(u)=\frac{1}{2}-\theta Beta(2,\beta +1){\left(1-u\right)}^{\alpha -1}[1-(1+\alpha )u],$$

where *Beta*(·,·) is the beta function defined by $Beta(a,b)=\int_{0}^{1}t^{a-1}{\left(1-t\right)}^{b-1}$ for *a, b *> 0.

By considering the proportion of variation for the directional dependence, two types of measure can be derived. From (2) with *k *= 2, we have

$$\begin{array}{lll} \rho_{U\to V}^{\left(2\right)} & = & 12\theta^{2}{\left[Beta(2,\beta +1)\right]}^{2}(Beta(1,2\alpha -1)\\ & & -2(1+\alpha )Beta(2,2\alpha -1)+{\left(1+\alpha \right)}^{2}Beta(3,2\alpha -1)),\text{ and}\\ \rho_{V\to U}^{\left(2\right)} & = & 12\theta^{2}[Beta(2,\alpha +1)]2(Beta(1,2\beta -1)\\ & & -2(1+\beta )Beta(2,2\beta -1)+{\left(1+\beta \right)}^{2}Beta(3,2\beta -1)), \end{array}$$

where Spearman's correlation coefficient, *ρ*_*c*_, is

$$\rho_{c}=12\int_{0}^{1}\int_{0}^{1}(C(u,v)-uv)\;dudv=12\theta \;Beta(2,\alpha +1)Beta(2,\beta +1).$$

Also, the following is a good case of extracting dependence information from a bivariate normal distribution function with respect to *ρ*. The relation is

$$\frac{\partial \Phi (x,y;\rho )}{\partial \rho }=\varphi (x,y;\rho )$$

where

$$\begin{array}{ccc} \varphi (x,y;\rho ) & = & \frac{1}{2\pi \sqrt{1-\rho^{2}}}exp\left\{-\frac{x^{2}-2\rho xy+y^{2}}{2(1-\rho^{2})}\right\} \end{array}$$

and

$$\Phi (x,y;\rho )=\int_{-\infty }^{x}\int_{\infty }^{y}\varphi (u,v;\rho )dudv.$$

We consider a parameterized copula which has θ_* _*ϕ*(Φ^-1 ^(*u*), Φ^-1 ^(*v*); α θ_*_) instead of *θ uv*(1 - *u*)^*α *^(1 - *v*)^*β *^at (3).

The form is as follows:

(6) *C*(*u, v*) = *uv *+ θ_* _*ϕ*(Φ^-1 ^(*u*), Φ^-1 ^(*v*); α θ_*_) for 0 ≤ *u, v *≤ 1,

where θ_* _is a parameter, *α *satisfies the following relation

$$\begin{array}{ccc} \varphi (x,y;\theta_{*}) & = & \varphi (x,y;\alpha \theta_{*})\left\{1+\frac{\alpha \theta_{*}}{1-\alpha^{2}\theta_{*}^{2}}\left[\alpha \theta_{*}(1-\alpha^{2}\theta_{*}^{2})+xy(1+\alpha^{2}\theta_{*}^{2})-\alpha \theta_{*}(x^{2}+y^{2})\right]\right\}, \end{array}$$

and

$$\varphi (x,y;\theta_{*})=\frac{1}{2\pi \sqrt{1-\theta_{*}^{2}}}exp\left\{-\frac{x^{2}-2\theta_{*}xy+y^{2}}{2(1-\theta_{*}^{2})}\right\}$$

¿From (6), the form of Spearman's correlation coefficient is

(7) $$\rho_{norm}=12\int_{0}^{1}\int_{0}^{1}C(u,v)dudv-3=\frac{6}{\pi }arcsin\left(\frac{\theta_{*}}{2}\right).$$

We use Akaike's information criterion (AIC) [24] for copula defined as

*AIC *= -2 log *L *(*θ*; **u**, **v**) + 2*υ*,

where *υ *is the number of parameters of the model provided in [25]. Akaike developed a decision-making strategy based on the Kullback-Leibler information measure, arguing that his measure provides a natural criterion for ordering alternative statistical models for data [24]. Instead of comparing plots or p-values for the methods, in the case of the parametric approach of maximum likelihood, we can compare the value of the negative log-likelihood functions. The value of AIC contains the information which estimator fits better. The lower the AIC, the better the model.

## Authors' contributions

J–MK proposed the research project and wrote the manuscript. Y–SJ performed statistical analysis. EAS supported the directional dependence with copula. K–HH performed the biological analysis and wrote a part of the manuscript. CP supported the research and IS designed the experiments. All authors read and approved the final manuscript.

## Supplementary Material

Additional file 1 Parameter estimates for the directional dependence at Group II. The multi-page table provides the estimates of *α, β, θ *and proportions of variation for the directional dependence at Group II. Click here for file

Additional file 2 Direct experimental support for the interactions uncovered. The multi-page table shows direct experimental support for the interactions uncovered. Click here for file
