# Supplementary material for: A copula method for modeling directional dependence of genes
Source: BMC Bioinformatics. 2008 May 1;9:225. doi: 10.1186/1471-2105-9-225 (PMC2386493; doi:10.1186/1471-2105-9-225)
Supplement: Additional file 1 — Parameter estimates for the directional dependence at Group II. The multi-page table provides the estimates of α, β, θ and proportions of variation for the directional dependence at Group II. [file 1471-2105-9-225-S1.pdf]

Interacting genes	AIC	FGM type						Normal Type	
		$\widehat{\alpha}$	$\widehat{\beta}$	$\widehat{\theta}$	ρ_C^2	$\rho_{U\to V}^{(2)}$	$\rho_{V\to U}^{(2)}$	$\widehat{\theta}_*$	ρ_{norm}^2
CLB5 vs CLB6	-27.9694	1.02152	1.01389	1.0716	0.12030	0.12037	0.12033	0.159	0.02310
CLB5 vs PRI2	-17.9198	1.02152	1.02216	1.1027	0.12565	0.12573	0.12574	0.285	0.07457
CLB5 vs POL12	-19.4287	1.02152	1.02184	1.1027	0.12572	0.12580	0.12580	0.091	0.00755
CLB5 vs PRI1	-0.4337	1.02152	1.02015	0.7961	0.06571	0.06575	0.06574	0.426	0.16805
CLB5 vs RAD53	-24.5830	1.02152	1.01903	1.0928	0.12405	0.12413	0.12411	0.090	0.00739
CLB5 vs POL30	-21.0601	1.02152	1.02219	1.1027	0.12565	0.12573	0.12573	0.293	0.07885
CLB5 vs MCD1	-10.3149	1.02152	1.02570	1.1027	0.12492	0.12500	0.12503	0.816	0.64423
CLB5 vs MSH2	-19.0271	1.02152	1.02001	1.0967	0.12473	0.12481	0.12480	0.703	0.47055
CLB5 vs RFA3	-3.3637	1.02152	1.02146	1.0193	0.10749	0.10756	0.10756	0.184	0.03096
CLB5 vs MSH6	-17.6580	1.02152	1.02136	1.1020	0.12566	0.12574	0.12574	0.699	0.46497
CLB5 vs CDC45	-18.5776	1.02152	1.02165	1.1027	0.12576	0.12584	0.12584	0.927	0.84720
CLB5 vs PMS1	-26.9607	1.02152	1.02133	1.1019	0.12564	0.12572	0.12572	0.356	0.11681
CLB5 vs PDS1	-24.0169	1.02152	1.02320	1.1027	0.12544	0.12552	0.12553	0.728	0.50628
CLB5 vs POL1	-5.5110	1.02152	1.02229	1.1027	0.12563	0.12570	0.12571	0.528	0.26035
CLB5 vs ASF1	-16.3535	1.02152	1.02190	1.1027	0.12738	0.12746	0.12742	0.812	0.63753
CLB5 vs RAD54	-20.6449	1.02152	1.02134	1.1020	0.12550	0.12557	0.12558	0.605	0.34448
CLB5 vs POL2	-16.7379	1.02152	1.01742	1.0863	0.12201	0.12208	0.12209	0.777	0.58070
CLB5 vs HPR5	-3.9288	1.02152	1.01993	1.0460	0.11344	0.11351	0.11350	0.600	0.33863
CLB6 vs PRI2	-20.2845	1.01389	1.02216	1.0716	0.12017	0.12020	0.12025	0.097	0.00858
CLB6 vs POL12	-17.0243	1.01389	1.02184	1.0716	0.12024	0.12027	0.12031	0.314	0.09065
CLB6 vs PRI1	3.7788	1.01389	1.02015	0.5076	0.02705	0.02706	0.02706	0.796	0.61116
CLB6 vs RAD53	-26.5287	1.01389	1.01903	1.0716	0.12080	0.12083	0.12085	0.886	0.76827
CLB6 vs POL30	-15.0167	1.01389	1.02219	1.0716	0.12017	0.12020	0.12025	0.563	0.29701
CLB6 vs MCD1	-4.7936	1.01389	1.02570	0.9383	0.09160	0.09162	0.09168	0.720	0.49468
CLB6 vs MSH2	-12.5225	1.01389	1.02001	1.0716	0.12060	0.12063	0.12066	0.866	0.73148
CLB6 vs RFA3	3.5023	1.01389	1.02146	0.5491	0.03159	0.03159	0.03161	0.374	0.12906
CLB6 vs MSH6	-14.3759	1.01389	1.02136	1.0716	0.12033	0.12036	0.12040	0.454	0.19127
CLB6 vs CDC45	-15.4106	1.01389	1.02165	1.0716	0.12027	0.12030	0.12035	0.700	0.46636
CLB6 vs PMS1	-21.9463	1.01389	1.02133	1.0716	0.12034	0.12037	0.12041	0.932	0.85716
CLB6 vs PDS1	-12.4043	1.01389	1.02320	1.0716	0.11997	0.12000	0.12005	0.507	0.23960
CLB6 vs POL1	-2.1922	1.01389	1.02229	0.9757	0.09960	0.09963	0.09967	0.870	0.73875
CLB6 vs ASF1	-14.6252	1.01389	1.02190	1.0716	0.11928	0.11936	0.11934	0.859	0.71886
CLB6 vs RAD54	-13.6652	1.01389	1.02134	1.0716	0.11866	0.11873	0.11874	0.635	0.38076
CLB6 vs POL2	-13.3911	1.01389	1.01742	1.0716	0.11798	0.11805	0.11808	0.258	0.06103
CLB6 vs HPR5	-4.7119	1.01389	1.01993	1.0050	0.10475	0.10481	0.10480	0.722	0.49757

Interacting genes	AIC	FGM type						Normal Type	
		$\widehat{\alpha}$	$\widehat{\beta}$	$\widehat{\theta}$	ρ_C^2	$\rho_{U\to V}^{(2)}$	$\rho_{V\to U}^{(2)}$	$\widehat{\theta}_*$	ρ_{norm}^2
PRI2 vs POL12	-17.4998	1.02216	1.02184	1.1039	0.12586	0.12594	0.12594	0.151	0.02083
PRI2 vs PRI1	-8.7171	1.02216	1.02015	1.0973	0.12471	0.12479	0.12477	0.933	0.85916
PRI2 vs RAD53	-24.1731	1.02216	1.01903	1.0928	0.12392	0.12400	0.12398	0.618	0.35995
PRI2 vs POL30	-27.1013	1.02216	1.02219	1.1052	0.12609	0.12617	0.12617	0.443	0.18196
PRI2 vs MCD1	-21.1117	1.02216	1.02570	1.1052	0.12536	0.12544	0.12547	0.136	0.01689
PRI2 vs MSH2	-25.1967	1.02216	1.02001	1.0967	0.12460	0.12468	0.12467	0.324	0.09657
PRI2 vs RFA3	-9.7898	1.02216	1.02146	1.1024	0.12560	0.12568	0.12567	0.865	0.72967
PRI2 vs MSH6	-25.2445	1.02216	1.02136	1.1020	0.12553	0.12561	0.12560	0.571	0.30576
PRI2 vs CDC45	-25.6714	1.02216	1.02165	1.1032	0.12574	0.12582	0.12582	0.598	0.33630
PRI2 vs PMS1	-26.4860	1.02216	1.02133	1.1019	0.12551	0.12559	0.12559	0.669	0.42432
PRI2 vs PDS1	-4.5684	1.02216	1.02320	1.1052	0.12588	0.12596	0.12596	0.516	0.24838
PRI2 vs POL1	-6.4101	1.02216	1.02229	1.1052	0.12606	0.12615	0.12615	0.730	0.50920
PRI2 vs ASF1	-27.5483	1.02216	1.02190	1.1041	0.12612	0.12620	0.12620	0.986	0.96943
PRI2 vs RAD54	-8.1826	1.02216	1.02134	1.1020	0.12566	0.12574	0.12574	0.698	0.46358
PRI2 vs POL2	-18.0154	1.02216	1.01742	1.0863	0.12205	0.12212	0.12212	0.836	0.67833
PRI2 vs HPR5	2.4296	1.02216	1.01993	0.6523	0.04403	0.04405	0.04405	0.837	0.68007
POL12 vs PRI1	-4.8053	1.02184	1.02015	1.0420	0.11251	0.11259	0.11257	0.161	0.02368
POL12 vs RAD53	-24.0722	1.02184	1.01903	1.0928	0.12398	0.12406	0.12404	0.188	0.03232
POL12 vs POL30	-25.4612	1.02184	1.02219	1.1039	0.12586	0.12594	0.12594	0.583	0.31914
POL12 vs MCD1	-19.3522	1.02184	1.02570	1.1039	0.12513	0.12521	0.12524	0.370	0.12628
POL12 vs MSH2	-26.1530	1.02184	1.02001	1.0967	0.12467	0.12475	0.12473	0.077	0.00540
POL12 vs RFA3	0.7029	1.02184	1.02146	0.7558	0.05907	0.05910	0.05910	0.275	0.06940
POL12 vs MSH6	-24.4460	1.02184	1.02136	1.1020	0.12560	0.12567	0.12567	0.318	0.09300
POL12 vs CDC45	-25.8463	1.02184	1.02165	1.1032	0.12581	0.12589	0.12589	0.970	0.93524
POL12 vs PMS1	-23.9932	1.02184	1.02133	1.1019	0.12558	0.12566	0.12565	0.215	0.04231
POL12 vs PDS1	-17.0151	1.02184	1.02320	1.1039	0.12565	0.12573	0.12574	0.925	0.84324
POL12 vs POL1	-8.5678	1.02184	1.02229	1.1039	0.12583	0.12591	0.12592	0.744	0.52993
POL12 vs ASF1	-25.0132	1.02184	1.02190	1.1039	0.12571	0.12579	0.12580	0.791	0.60306
POL12 vs RAD54	-18.7044	1.02184	1.02134	1.1020	0.12547	0.12554	0.12555	0.478	0.21244
POL12 vs POL2	-25.2960	1.02184	1.01742	1.0863	0.12200	0.12207	0.12207	0.361	0.12015
POL12 vs HPR5	-0.3169	1.02184	1.01993	0.8522	0.07515	0.07520	0.07519	0.549	0.28203
PRI1 vs RAD53	-0.8503	1.02015	1.01903	0.9313	0.09030	0.09034	0.09034	0.603	0.34213
PRI1 vs POL30	-13.7608	1.02015	1.02219	1.0973	0.12470	0.12477	0.12478	0.473	0.20793
PRI1 vs MCD1	-9.9099	1.02015	1.02570	1.0973	0.12398	0.12405	0.12409	0.057	0.00296
PRI1 vs MSH2	-19.6331	1.02015	1.02001	1.0967	0.12502	0.12508	0.12508	0.040	0.00145
PRI1 vs RFA3	-15.8811	1.02015	1.02146	1.0973	0.12485	0.12492	0.12493	0.347	0.11091

Interacting genes	AIC	FGM type						Normal Type	
		$\widehat{\alpha}$	$\widehat{\beta}$	$\widehat{\theta}$	ρ^2_C	$\rho^{(2)}_{U\to V}$	$\rho^{(2)}_{V\to U}$	$\widehat{\theta}_*$	ρ^2_{norm}
PRI1 vs MSH6	-11.1239	1.02015	1.02136	1.0973	0.12487	0.12494	0.12495	0.682	0.44168
PRI1 vs CDC45	-8.4673	1.02015	1.02165	1.0973	0.12481	0.12488	0.12489	0.066	0.00397
PRI1 vs PMS1	-9.1108	1.02015	1.02133	1.0973	0.12488	0.12495	0.12495	0.316	0.09182
PRI1 vs PDS1	-2.6663	1.02015	1.02320	0.8746	0.07909	0.07913	0.07914	0.216	0.04271
PRI1 vs POL1	-8.7023	1.02015	1.02229	1.0973	0.12468	0.12475	0.12476	0.121	0.01336
PRI1 vs ASF1	-16.3147	1.02015	1.02190	1.0973	0.12541	0.12548	0.12546	0.663	0.41644
PRI1 vs RAD54	-6.1673	1.02015	1.02134	1.0928	0.12386	0.12394	0.12393	0.578	0.31352
PRI1 vs POL2	-12.1890	1.02015	1.01742	1.0863	0.12349	0.12352	0.12357	0.611	0.35157
PRI1 vs HPR5	5.8687	1.02015	1.01993	0.1172	0.00143	0.00143	0.00143	0.287	0.07563
RAD53 vs POL30	-25.2215	1.01903	1.02219	1.0928	0.12391	0.12397	0.12399	0.293	0.07885
RAD53 vs MCD1	-17.8648	1.01903	1.02570	1.0928	0.12320	0.12326	0.12330	0.273	0.06838
RAD53 vs MSH2	-23.6906	1.01903	1.02001	1.0928	0.12436	0.12442	0.12442	0.616	0.35755
RAD53 vs RFA3	-0.0257	1.01903	1.02146	0.7904	0.06490	0.06493	0.06494	0.145	0.01920
RAD53 vs MSH6	-22.8629	1.01903	1.02136	1.0928	0.12408	0.12414	0.12416	0.950	0.89361
RAD53 vs CDC45	-26.4966	1.01903	1.02165	1.0928	0.12402	0.12408	0.12410	0.504	0.23671
RAD53 vs PMS1	-24.3796	1.01903	1.02133	1.0928	0.12409	0.12415	0.12416	0.827	0.66286
RAD53 vs PDS1	-12.3134	1.01903	1.02320	1.0928	0.12371	0.12377	0.12379	0.310	0.08834
RAD53 vs POL1	-9.2658	1.01903	1.02229	1.0928	0.12389	0.12395	0.12397	0.394	0.14342
RAD53 vs ASF1	-19.3311	1.01903	1.02190	1.0928	0.12539	0.12542	0.12546	0.209	0.03997
RAD53 vs RAD54	-12.5997	1.01903	1.02134	1.0928	0.12562	0.12566	0.12568	0.670	0.42564
RAD53 vs POL2	-26.3996	1.01903	1.01742	1.0863	0.12349	0.12352	0.12357	0.854	0.70993
RAD53 vs HPR5	-0.8166	1.01903	1.01993	0.8218	0.07026	0.07028	0.07032	0.116	0.01228
POL30 vs MCD1	-25.2519	1.02219	1.02570	1.1053	0.12538	0.12546	0.12548	0.173	0.02736
POL30 vs MSH2	-31.3348	1.02219	1.02001	1.0967	0.12460	0.12468	0.12466	0.028	0.00071
POL30 vs RFA3	-18.4140	1.02219	1.02146	1.1024	0.12559	0.12567	0.12567	0.457	0.19385
POL30 vs MSH6	-29.7512	1.02219	1.02136	1.1020	0.12552	0.12560	0.12560	0.626	0.36966
POL30 vs CDC45	-25.4694	1.02219	1.02165	1.1032	0.12574	0.12582	0.12581	0.316	0.09182
POL30 vs PMS1	-28.5919	1.02219	1.02133	1.1019	0.12551	0.12559	0.12558	0.792	0.60468
POL30 vs PDS1	-15.9145	1.02219	1.02320	1.1053	0.12589	0.12597	0.12598	0.792	0.60468
POL30 vs POL1	-14.1626	1.02219	1.02229	1.1053	0.12608	0.12616	0.12616	0.953	0.89978
POL30 vs ASF1	-23.3615	1.02219	1.02190	1.1041	0.12803	0.12806	0.12809	0.594	0.33168
POL30 vs RAD54	-13.4762	1.02219	1.02134	1.1020	0.12724	0.12727	0.12731	0.500	0.23288
POL30 vs POL2	-24.5341	1.02219	1.01742	1.0863	0.12366	0.12369	0.12373	0.212	0.04113
POL30 vs HPR5	1.2039	1.02219	1.01993	0.7691	0.06195	0.06197	0.06199	0.073	0.00486

Interacting genes	AIC	FGM type						Normal Type	
		$\widehat{\alpha}$	$\widehat{\beta}$	$\widehat{\theta}$	ρ_C^2	$\rho_{U\to V}^{(2)}$	$\rho_{V\to U}^{(2)}$	$\widehat{\theta}_*$	ρ_{norm}^2
MCD1 vs MSH2	-28.9162	1.02570	1.02001	1.0967	0.12388	0.12398	0.12394	0.030	0.00082
MCD1 vs RFA3	-12.2984	1.02570	1.02146	1.1024	0.12487	0.12498	0.12494	0.630	0.37457
MCD1 vs MSH6	-32.7523	1.02570	1.02136	1.1020	0.12480	0.12491	0.12487	0.770	0.56970
MCD1 vs CDC45	-27.2708	1.02570	1.02165	1.1032	0.12501	0.12512	0.12509	0.498	0.23098
MCD1 vs PMS1	-19.0614	1.02570	1.02133	1.1019	0.12478	0.12489	0.12486	0.754	0.54503
MCD1 vs PDS1	-5.23520	1.02570	1.02320	0.9964	0.10172	0.10180	0.10179	0.573	0.30797
MCD1 vs POL1	-8.88663	1.02570	1.02229	1.1057	0.12544	0.12555	0.12553	0.344	0.10899
MCD1 vs ASF1	-23.4712	1.02570	1.02190	1.1041	0.12775	0.12778	0.12782	0.233	0.04973
MCD1 vs RAD54	-4.5810	1.02570	1.02134	1.0012	0.10472	0.10475	0.10480	0.204	0.03808
MCD1 vs POL2	-21.7087	1.02570	1.01742	1.0863	0.12347	0.12350	0.12355	0.763	0.55883
MCD1 vs HPR5	5.7209	1.02570	1.01993	0.1823	0.00347	0.00348	0.00348	0.217	0.04310
MSH2 vs RFA3	-18.3638	1.02001	1.02146	1.0967	0.12475	0.12481	0.12482	0.378	0.13187
MSH2 vs MSH6	-32.4131	1.02001	1.02136	1.0967	0.12477	0.12483	0.12484	0.322	0.09537
MSH2 vs CDC45	-24.7471	1.02001	1.02165	1.0967	0.12471	0.12477	0.12478	0.129	0.01519
MSH2 vs PMS1	-25.5087	1.02001	1.02133	1.0967	0.12477	0.12484	0.12485	0.124	0.01403
MSH2 vs PDS1	-18.9586	1.02001	1.02320	1.0967	0.12439	0.12445	0.12448	0.581	0.31688
MSH2 vs POL1	-15.4200	1.02001	1.02229	1.0967	0.12457	0.12464	0.12466	0.856	0.71350
MSH2 vs ASF1	-26.7082	1.02001	1.02190	1.0967	0.12604	0.12607	0.12611	0.555	0.28840
MSH2 vs RAD54	-14.3371	1.02001	1.02134	1.0967	0.12686	0.12689	0.12691	0.937	0.86719
MSH2 vs POL2	-31.0431	1.02001	1.01742	1.0863	0.12395	0.12398	0.12401	0.929	0.85118
MSH2 vs HPR5	0.8542	1.02001	1.01993	0.8093	0.06765	0.06769	0.06769	0.088	0.00706
RFA3 vs MSH6	-14.7987	1.02146	1.02136	1.1020	0.12567	0.12575	0.12575	0.951	0.89566
RFA3 vs CDC45	-6.9921	1.02146	1.02165	1.1024	0.12570	0.12578	0.12578	0.061	0.00339
RFA3 vs PMS1	-4.6701	1.02146	1.02133	1.1019	0.12566	0.12573	0.12573	0.774	0.57597
RFA3 vs PDS1	-8.6447	1.02146	1.02320	1.0555	0.11494	0.11501	0.11502	0.220	0.04431
RFA3 vs POL1	-6.3768	1.02146	1.02229	1.1002	0.12507	0.12515	0.12515	0.276	0.06990
RFA3 vs ASF1	-7.1144	1.02146	1.02190	1.1024	0.12587	0.12595	0.12594	0.594	0.33168
RFA3 vs RAD54	1.8378	1.02146	1.02134	0.6455	0.04323	0.04326	0.04325	0.105	0.01006
RFA3 vs POL2	-10.8199	1.02146	1.01742	1.0863	0.12181	0.12189	0.12189	0.124	0.01403
RFA3 vs HPR5	2.7166	1.02146	1.01993	0.6171	0.03908	0.03910	0.03911	0.552	0.28521
MSH6 vs CDC45	-26.0668	1.02136	1.02165	1.1020	0.12563	0.12571	0.12571	0.834	0.67488
MSH6 vs PMS1	-26.0289	1.02136	1.02133	1.1019	0.12568	0.12575	0.12575	0.091	0.00755
MSH6 vs PDS1	-8.4754	1.02136	1.02320	1.0223	0.10784	0.10791	0.10792	0.949	0.89156
MSH6 vs POL1	-11.5811	1.02136	1.02229	1.1020	0.12550	0.12558	0.12558	0.073	0.00486
MSH6 vs ASF1	-27.3734	1.02136	1.02190	1.1020	0.12581	0.12589	0.12587	0.774	0.57597
MSH6 vs RAD54	-9.6066	1.02136	1.02134	1.0455	0.11297	0.11304	0.11304	0.082	0.00613

Interacting genes	AIC	FGM type						Normal Type	
		$\widehat{\alpha}$	$\widehat{\beta}$	$\widehat{\theta}$	ρ_C^2	$\rho_{U\to V}^{(2)}$	$\rho_{V\to U}^{(2)}$	$\widehat{\theta}_*$	ρ_{norm}^2
MSH6 vs POL2	-29.0104	1.02136	1.01742	1.0863	0.12198	0.12206	0.12205	0.818	0.64759
MSH6 vs HPR5	3.6305	1.02136	1.01993	0.5462	0.03082	0.03084	0.03084	0.762	0.55729
CDC45 vs PMS1	-25.0628	1.02165	1.02133	1.1019	0.12562	0.12569	0.12569	0.821	0.65266
CDC45 vs PDS1	-10.5691	1.02165	1.02320	1.1032	0.12553	0.12560	0.12561	0.941	0.87526
CDC45 vs POL1	-11.6134	1.02165	1.02229	1.1032	0.12571	0.12579	0.12580	0.292	0.07831
CDC45 vs ASF1	-25.1085	1.02165	1.02190	1.1032	0.12581	0.12589	0.12588	0.224	0.04594
CDC45 vs RAD54	-8.1071	1.02165	1.02134	1.0646	0.11680	0.11687	0.11688	0.954	0.90184
CDC45 vs POL2	-21.8812	1.02165	1.01742	1.0863	0.12179	0.12187	0.12187	0.962	0.91844
CDC45 vs HPR5	0.0234	1.02165	1.01993	0.7694	0.06113	0.06117	0.06117	0.784	0.59182
PMS1 vs PDS1	-13.3989	1.02133	1.02320	1.1019	0.12530	0.12537	0.12538	0.048	0.00210
PMS1 vs POL1	-13.8295	1.02133	1.02229	1.1019	0.12548	0.12556	0.12557	0.951	0.89566
PMS1 vs ASF1	-24.5612	1.02133	1.02190	1.1019	0.12551	0.12559	0.12558	0.794	0.60792
PMS1 vs RAD54	-17.9898	1.02133	1.02134	1.1019	0.12633	0.12641	0.12638	0.526	0.25833
PMS1 vs POL2	-20.7906	1.02133	1.01742	1.0863	0.12227	0.12234	0.12233	0.079	0.00569
PMS1 vs HPR5	-1.9078	1.02133	1.01993	1.0044	0.10454	0.10461	0.10460	0.961	0.91636
PDS1 vs POL1	-3.2131	1.02320	1.02229	0.8970	0.08290	0.08296	0.08295	0.650	0.39965
PDS1 vs ASF1	-8.8879	1.02320	1.02190	1.1041	0.12656	0.12664	0.12662	0.137	0.01714
PDS1 vs RAD54	-18.1267	1.02320	1.02134	1.1020	0.12542	0.12550	0.12550	0.216	0.04271
PDS1 vs POL2	-13.0286	1.02320	1.01742	1.0863	0.12117	0.12125	0.12128	0.188	0.03232
PDS1 vs HPR5	-8.2617	1.02320	1.01993	1.0964	0.12460	0.12468	0.12467	0.052	0.00246
POL1 vs ASF1	-13.4642	1.02229	1.02190	1.1041	0.12605	0.12613	0.12613	0.760	0.55421
POL1 vs RAD54	-0.9813	1.02229	1.02134	0.8430	0.07349	0.07354	0.07354	0.610	0.35039
POL1 vs POL2	-17.4798	1.02229	1.01742	1.0863	0.12198	0.12206	0.12206	0.736	0.51803
POL1 vs HPR5	4.0675	1.02229	1.01993	0.4781	0.02364	0.02365	0.02365	0.114	0.01186
ASD1 vs RAD54	-17.3026	1.02190	1.02134	1.1020	0.12521	0.12529	0.12530	0.350	0.11286
ASF1 vs POL2	-25.0023	1.02190	1.01742	1.0863	0.12185	0.12193	0.12193	0.618	0.35995
ASF1 vs HPR5	5.9782	1.02190	1.01993	0.0515	0.00027	0.00027	0.00027	0.934	0.86116
RAD54 vs POL2	-12.7519	1.02134	1.01742	1.0863	0.12205	0.12212	0.12212	0.515	0.24739
RAD54 vs HPR5	1.0450	1.02134	1.01993	0.7242	0.05459	0.05463	0.05461	0.708	0.47758
POL2 vs HPR5	2.2370	1.01742	1.01993	0.6541	0.04435	0.04438	0.04437	0.474	0.20883
