# Supplementary material for: A copula method for modeling directional dependence of genes
Source: BMC Bioinformatics. 2008 May 1;9:225. doi: 10.1186/1471-2105-9-225 (PMC2386493; doi:10.1186/1471-2105-9-225)
Supplement: Additional file 2 — Direct experimental support for the interactions uncovered. The multi-page table shows direct experimental support for the interactions uncovered. [file 1471-2105-9-225-S2.pdf]

Interacting genes (locus name)	Our Method	PathwayAssist	Chen's method
POL30(YBR088C)-CLB6(YGR109C)	O	×	×
POL30(YBR088C)-PRI2(YKL045W)	O	×	×
POL30(YBR088C)-POL12(YBL035C)	O	×	×
POL30(YBR088C)-PRI1(YIR008C)	O	×	×
POL30(YBR088C)-RAD53(YPL153C)	O	×	×
POL30(YBR088C)-CLB5(YPR120C)	O	×	O
POL30(YBR088C)-MCD1(YDL003W)	O	O	O
POL30(YBR088C)-MSH2(YOL090W)	O	×	×
POL30(YBR088C)-RFA3(YJL173C)	O	×	×
POL30(YBR088C)-MSH6(YDR097C)	O	×	×
POL30(YBR088C)-CDC45(YLR103C)	O	×	×
POL30(YBR088C)-PMS1(YNL082W)	O	×	×
POL30(YBR088C)-PDS1(YDR113C)	O	×	×
POL30(YBR088C)-POL1(YNL102W)	O	×	O
POL30(YBR088C)-ASF1(YJL115W)	O	×	×
POL30(YBR088C)-RAD54(YGL163C)	O	×	×
POL30(YBR088C)-POL2(YNL262W)	O	×	×
POL30(YBR088C)-HPR5(YJL092W)	O	×	×
MCD1(YDL003W)-CLB6(YGR109C)	O	×	×
MCD1(YDL003W)-PRI2(YKL045W)	O	×	O
MCD1(YDL003W)-POL12(YBL035C)	O	×	×
MCD1(YDL003W)-PRI1(YIR008C)	O	×	×
MCD1(YDL003W)-RAD53(YPL153C)	O	×	×
MCD1(YDL003W)-CLB5(YPR120C)	O	×	×
MCD1(YDL003W)-MSH2(YOL090W)	O	×	O
MCD1(YDL003W)-RFA3(YJL173C)	O	×	×
MCD1(YDL003W)-MSH6(YDR097C)	O	×	×
MCD1(YDL003W)-CDC45(YLR103C)	O	×	O
MCD1(YDL003W)-PMS1(YNL082W)	O	×	O
MCD1(YDL003W)-PDS1(YDR113C)	O	×	×
MCD1(YDL003W)-POL1(YNL102W)	O	×	×
MCD1(YDL003W)-ASF1(YJL115W)	O	×	O
MCD1(YDL003W)-RAD54(YGL163C)	O	×	×
MCD1(YDL003W)-POL2(YNL262W)	O	×	×
MCD1(YDL003W)-HPR5(YJL092W)	O	×	×
PRI2(YKL045W)-CLB6(YGR109C)	O	×	×
PRI2(YKL045W)-POL12(YBL035C)	O	O	O

Interacting genes (locus name)	Our Method	PathwayAssist	Chen's method
PRI2(YKL045W)-PRI1(YIR008C)	O	×	×
PRI2(YKL045W)-RAD53(YPL153C)	O	×	×
PRI2(YKL045W)-MSH2(YOL090W)	O	×	×
PRI2(YKL045W)-RFA3(YJL173C)	O	×	×
PRI2(YKL045W)-MSH6(YDR097C)	O	×	O
PRI2(YKL045W)-CDC45(YLR103C)	O	×	×
PRI2(YKL045W)-PMS1(YNL082W)	O	×	×
PRI2(YKL045W)-PDS1(YDR113C)	O	×	×
PRI2(YKL045W)-POL1(YNL102W)	O	×	×
PRI2(YKL045W)-ASF1(YJL115W)	O	×	O
PRI2(YKL045W)-RAD54(YGL163C)	O	×	×
PRI2(YKL045W)-POL2(YNL262W)	O	×	×
PRI2(YKL045W)-HPR5(YJL092W)	O	×	×
PRI2(YKL045W)-CLB5(YPR120C)	O	×	×
POL1(YNL102W)-CLB6(YGR109C)	O	×	×
POL1(YNL102W)-POL12(YBL035C)	O	×	×
POL1(YNL102W)-PRI1(YIR008C)	O	×	×
POL1(YNL102W)-RAD53(YPL153C)	O	×	×
POL1(YNL102W)-MSH2(YOL090W)	O	×	O
POL1(YNL102W)-RFA3(YJL173C)	O	×	×
POL1(YNL102W)-MSH6(YDR097C)	O	×	×
POL1(YNL102W)-CDC45(YLR103C)	O	×	O
POL1(YNL102W)-PMS1(YNL082W)	O	×	×
POL1(YNL102W)-PDS1(YDR113C)	O	×	×
POL1(YNL102W)-ASF1(YJL115W)	O	×	O
POL1(YNL102W)-RAD54(YGL163C)	O	×	×
POL1(YNL102W)-POL2(YNL262W)	O	×	×
POL1(YNL102W)-HPR5(YJL092W)	O	×	×
POL1(YNL102W)-CLB5(YPR120C)	O	×	×
MSH2(YOL090W)-CLB6(YGR109C)	O	×	×
MSH2(YOL090W)-POL12(YBL035C)	O	×	×
MSH2(YOL090W)-PRI1(YIR008C)	O	×	O
MSH2(YOL090W)-RAD53(YPL153C)	O	×	×
MSH2(YOL090W)-RFA3(YJL173C)	O	O	O
MSH2(YOL090W)-MSH6(YDR097C)	O	O	O
MSH2(YOL090W)-CDC45(YLR103C)	O	×	×
MSH2(YOL090W)-PMS1(YNL082W)	O	×	×

Interacting genes (locus name)	Our Method	PathwayAssist	Chen's method
MSH2(YOL090W)-PDS1(YDR113C)	O	×	O
MSH2(YOL090W)-ASF1(YJL115W)	O	×	×
MSH2(YOL090W)-RAD54(YGL163C)	O	×	×
MSH2(YOL090W)-POL2(YNL262W)	O	×	×
MSH2(YOL090W)-HPR5(YJL092W)	O	×	×
MSH2(YOL090W)-CLB5(YPR120C)	O	×	×
POL12(YBL035C)-CLB6(YGR109C)	O	×	×
POL12(YBL035C)-PRI1(YIR008C)	O	×	×
POL12(YBL035C)-RAD53(YPL153C)	O	×	O
POL12(YBL035C)-RFA3(YJL173C)	O	×	×
POL12(YBL035C)-MSH6(YDR097C)	O	×	×
POL12(YBL035C)-CDC45(YLR103C)	O	×	O
POL12(YBL035C)-PMS1(YNL082W)	O	×	×
POL12(YBL035C)-PDS1(YDR113C)	O	×	×
POL12(YBL035C)-ASF1(YJL115W)	O	×	×
POL12(YBL035C)-RAD54(YGL163C)	O	×	×
POL12(YBL035C)-POL2(YNL262W)	O	×	×
POL12(YBL035C)-HPR5(YJL092W)	O	×	×
POL12(YBL035C)-CLB5(YPR120C)	O	×	×
ASF1(YJL115W)-CLB6(YGR109C)	O	×	×
ASF1(YJL115W)-PRI1(YIR008C)	O	×	×
ASF1(YJL115W)-RAD53(YPL153C)	O	×	×
ASF1(YJL115W)-RFA3(YJL173C)	O	×	×
ASF1(YJL115W)-MSH6(YDR097C)	O	×	O
ASF1(YJL115W)-CDC45(YLR103C)	O	×	×
ASF1(YJL115W)-PMS1(YNL082W)	O	×	×
ASF1(YJL115W)-PDS1(YDR113C)	O	×	×
ASF1(YJL115W)-RAD54(YGL163C)	O	×	O
ASF1(YJL115W)-POL2(YNL262W)	O	×	×
ASF1(YJL115W)-HPR5(YJL092W)	O	×	×
ASF1(YJL115W)-CLB5(YPR120C)	O	×	×
PRI1(YIR008C)-CLB6(YGR109C)	O	×	×
PRI1(YIR008C)-RAD53(YPL153C)	O	O	O
PRI1(YIR008C)-RFA3(YJL173C)	O	×	O
PRI1(YIR008C)-MSH6(YDR097C)	O	×	×
PRI1(YIR008C)-CDC45(YLR103C)	O	×	×
PRI1(YIR008C)-PMS1(YNL082W)	O	×	×

Interacting genes (locus name)	Our Method	PathwayAssist	Chen's method
PRI1(YIR008C)-PDS1(YDR113C)	O	×	O
PRI1(YIR008C)-RAD54(YGL163C)	O	×	×
PRI1(YIR008C)-POL2(YNL262W)	O	×	×
PRI1(YIR008C)-HPR5(YJL092W)	O	×	×
PRI1(YIR008C)-CLB5(YPR120C)	O	×	×
RFA3(YJL173C)-CLB6(YGR109C)	O	×	×
RFA3(YJL173C)-RAD53(YPL153C)	O	×	×
RFA3(YJL173C)-MSH6(YDR097C)	O	O	O
RFA3(YJL173C)-CDC45(YLR103C)	O	×	×
RFA3(YJL173C)-PMS1(YNL082W)	O	×	×
RFA3(YJL173C)-PDS1(YDR113C)	O	×	×
RFA3(YJL173C)-RAD54(YGL163C)	O	×	×
RFA3(YJL173C)-POL2(YNL262W)	O	×	×
RFA3(YJL173C)-HPR5(YJL092W)	O	×	×
RFA3(YJL173C)-CLB5(YPR120C)	O	×	×
PDS1(YDR113C)-CLB6(YGR109C)	O	×	×
PDS1(YDR113C)-RAD53(YPL153C)	O	×	×
PDS1(YDR113C)-MSH6(YDR097C)	O	×	×
PDS1(YDR113C)-CDC45(YLR103C)	O	×	×
PDS1(YDR113C)-PMS1(YNL082W)	O	×	×
PDS1(YDR113C)-RAD54(YGL163C)	O	×	×
PDS1(YDR113C)-POL2(YNL262W)	O	×	×
PDS1(YDR113C)-HPR5(YJL092W)	O	×	×
PDS1(YDR113C)-CLB5(YPR120C)	O	×	×
RAD54(YGL163C)-CLB6(YGR109C)	O	×	×
RAD54(YGL163C)-RAD53(YPL153C)	O	×	×
RAD54(YGL163C)-MSH6(YDR097C)	O	×	×
RAD54(YGL163C)-CDC45(YLR103C)	O	×	×
RAD54(YGL163C)-PMS1(YNL082W)	O	×	×
RAD54(YGL163C)-POL2(YNL262W)	O	×	O
RAD54(YGL163C)-HPR5(YJL092W)	O	×	×
RAD54(YGL163C)-CLB5(YPR120C)	O	×	×
MSH6(YDR097C)-CLB6(YGR109C)	O	×	×
MSH6(YDR097C)-RAD53(YPL153C)	O	×	×
MSH6(YDR097C)-CDC45(YLR103C)	O	×	O
MSH6(YDR097C)-PMS1(YNL082W)	O	×	×
MSH6(YDR097C)-POL2(YNL262W)	O	×	O

Interacting genes (locus name)	Our Method	PathwayAssist	Chen's method
MSH6(YDR097C)-HPR5(YJL092W)	O	×	×
MSH6(YDR097C)-CLB5(YPR120C)	O	×	×
CDC45(YLR103C)-CLB6(YGR109C)	O	×	×
CDC45(YLR103C)-RAD53(YPL153C)	O	×	O
CDC45(YLR103C)-PMS1(YNL082W)	O	×	×
CDC45(YLR103C)-POL2(YNL262W)	O	×	×
CDC45(YLR103C)-HPR5(YJL092W)	O	×	×
CDC45(YLR103C)-CLB5(YPR120C)	O	×	×
POL2(YNL262W)-CLB6(YGR109C)	O	×	×
POL2(YNL262W)-RAD53(YPL153C)	O	×	×
POL2(YNL262W)-PMS1(YNL082W)	O	×	×
POL2(YNL262W)-HPR5(YJL092W)	O	×	×
POL2(YNL262W)-CLB5(YPR120C)	O	×	×
RAD53(YPL153C)-CLB6(YGR109C)	O	×	×
RAD53(YPL153C)-PMS1(YNL082W)	O	×	O
RAD53(YPL153C)-HPR5(YJL092W)	O	×	×
RAD53(YPL153C)-CLB5(YPR120C)	O	×	O
HPR5(YJL092W)-CLB6(YGR109C)	O	×	×
HPR5(YJL092W)-PMS1(YNL082W)	O	×	×
HPR5(YJL092W)-CLB5(YPR120C)	O	×	O
CLB5(YPR120C)-CLB6(YGR109C)	O	O	O
CLB5(YPR120C)-PMS1(YNL082W)	O	×	×
PMS1(YNL082W)-CLB6(YGR109C)	O	×	×
